# Factors That Shape Women’s Physical Activity: Development of the Reasons to Participate in Physical Activity Scale (RPPAS)

**DOI:** 10.3390/healthcare10010094

**Published:** 2022-01-04

**Authors:** Zhaohui Su, Dean McDonnell, Ali Cheshmehzangi, Jing Zhu, Junaid Ahmad, Sabina Šegalo, Claudimar Pereira da Veiga

**Affiliations:** 1 Center on Smart and Connected Health Technologies, Mays Cancer Center, School of Nursing, UT Health San Antonio, San Antonio, TX 78229, USA; 2 Department of Humanities, Institute of Technology Carlow, R93 V960 Carlow, Ireland; dean.mcdonnell@itcarlow.ie; 3 Faculty of Science and Engineering, University of Nottingham Ningbo China, Ningbo 315100, China; Ali.Cheshmehzangi@nottingham.edu.cn; 4 Beijing Institute of Nutritional Resources, Beijing 100069, China; jingzhu.nutri@outlook.com; 5 Prime Institute of Public Health, Peshawar Medical College, Peshawar 25160, Pakistan; Jahmad@piph.prime.edu.pk; 6 Department of Microbiology, Faculty of Medicine, University of Sarajevo, 71000 Sarajevo, Bosnia and Herzegovina; sabina.segalo11@gmail.com; 7 School of Management—PPGOLD, Federal University of Parana—UFPR, Curitiba 80210-170, Brazil

**Keywords:** physical activity, women, factor analysis, obesity, health disparities, COVID-19, pandemic

## Abstract

(1) Background: Obesity could deepen women’s susceptibility to COVID-19 infections and deaths. While physical activity has the potential to improve women’s physical and psychological resilience to the pandemic, there is a dearth of research on factors that motivate women’s participation in physical activity. Thus, to bridge the research gap, this study aims to identify factors that motivate women’s participation in physical activity. (2) Methods: An online survey on motivations for physical activity was developed and distributed to the participants. A total of 108 women offered complete answers (*N* = 108, 18–33 years old, M_age_ = 20.34 ± 2.42 years). Participants selected factors that promote their physical activity from a list of 34 factors from the Reasons to Participate in Physical Activity Scale (RPPAS) developed in this study. (3) Results: Exploratory factor analysis revealed that factors that motivate women’s participation in physical activity are: enjoyment and gratification, consideration for other activities (i.e., exergaming), health benefits, networking opportunities, and appearance and performance. Multiple linear regression analyses indicate that only consideration for appearance and performance was significantly associated with participants’ physical activity levels after controlling for compounding factors. (4) Conclusions: The findings of this study underscore the importance of appearance and performance in shaping women’s participation in physical activity. Furthermore, the results also emphasize the need for a nuanced understanding of factors that influence women’s physical activity levels. Future research could investigate how to leverage these motivators in tailored health interventions that aim to improve women’s physical activity.

## 1. Background

A world without women is a world without a future. Women’s roles in childbearing, caregiving, and the workforce are indispensable to the integrity of society [1]. However, while the health of women shapes the wellbeing of humanity, women shoulder a wide array of health challenges that could substantially compromise their abilities to care for themselves and contribute to society [2]. Data suggest that, for instance, while 6.9% of men 20 years and older in the U.S. face severe obesity, 11.5% of women suffer the same condition [3], which means that women are more likely than their male counterparts to develop obesity-induced morbidity and mortality risks [4,5,6,7]. Take cancer, for instance: Research shows that while 25% of cancers diagnosed in men are overweight- and obesity-related cancers, the prevalence of these cancers among women is 55% [8]. COVID-19 could further compound the situation. In an analysis of 502,493 people (54% women), researchers found that a higher body mass index (BMI) is linked with greater risks of COVID-19 deaths in women than men [9]. In addition, researchers also found that the pandemic not only exacerbated the deep-rooted health disparities women face before the pandemic (e.g., limited access to healthcare services), it also introduced new challenges women need to shoulder, ranging from pronounced financial insecurity, an unprecedented surge of domestic violence, to health issues that are unique to women [10,11,12].

These insights combined, in turn, highlight the imperative to address the obesity epidemic in women, particularly amid COVID-19. One cost-effective approach that could reduce women’s susceptibility to health consequences of obesity, along with their vulnerability to COVID-19, is physical activity [13,14,15,16,17,18,19]. Physical activity broadly refers to the bodily movement of skeletal muscles that results in the expenditure of energy [20]. Mounting evidence suggests that physical activity is critical to people’s physical and mental health [21,22,23]. A longitudinal study conducted prior to the pandemic, for instance, shows that habitual physical activity positively impacts fat formation in adolescence and fat growth in adulthood [21], health improvements that are critical to maintaining physical health. Research conducted amid the pandemic further shows that physical activity can reduce people’s anxiety and boost their mood during COVID-19 [24], including periods of lockdowns [16,17,18,19].

However, though physical activity has great potential to help women fend off the adverse impacts of COVID-19 and beyond, women’s physical activity levels are suboptimal. A study of 1.9 million participants worldwide conducted prior to the pandemic shows that 31.7% of women, compared to 27.5% of men, have insufficient physical activity levels [25]. Analyses of data on 212,021 people from 51 countries further show that 20% of women, as opposed to 15% of men, are at heightened risks for chronic diseases due to physical inactivity [26], the gap which was confirmed by subsequent research [27]. The pandemic has further compounded the situation. Analyzing physical activity changes of 455,404 people from 187 countries between 19 January 2020 and 1 June 2020, researchers found that, though to varying degrees, the pandemic has reduced people’s physical activity levels across the world [24].

One way to boost women’s physical activity levels, as insights from value-expectancy theories [28,29,30,31] and persuasive communication literature [32,33,34] suggest, is via understanding what factors motivate women to participate in physical activity the most, and in turn, integrate these factors into tailored health interventions to boost women’s participation in physical activity. However, while useful insights are available, there is a lack of research on factors that motivate young females to improve their physical activity levels. Thus, to bridge the literature, this study aims to identify factors that motivate young women’s participation in physical activity that could help guard tailored intervention design and development.

## 2. Methods

### 2.1. Participants and Procedure

This study was approved by the University’s Institutional Review Board (IRB). It was part of a larger study that examines people’s physical activity behavior in light of technological alternatives such as exergaming (video-game-based exercise) [35]. Participants were recruited from Southwestern University’s large participant pool and were asked to read and agree to the consent form prior to participation. A written consent form was waived by the IRB office, and a digital variation was used. The survey was distributed online between March to May 2014, powered by the Qualtrics survey platform, and consists of both fixed and open-ended questions that examine people’s physical activity and exergaming behavior. 

Specifically, respondents were asked to report their sociodemographic background, factors that promote their physical activity, and leisure-time physical activity levels. All participants were informed regarding their rights prior to joining the study, including being able to withdraw from the research at any time without question. Detailed information on all the female and male participants’ sociodemographic and personal characteristics could be found in Table 1. Among all the respondents who participated in the study, we excluded those who did not self-identify as female and offered incomplete answers.

### 2.2. Development of Scale Items

**Figure 1:**
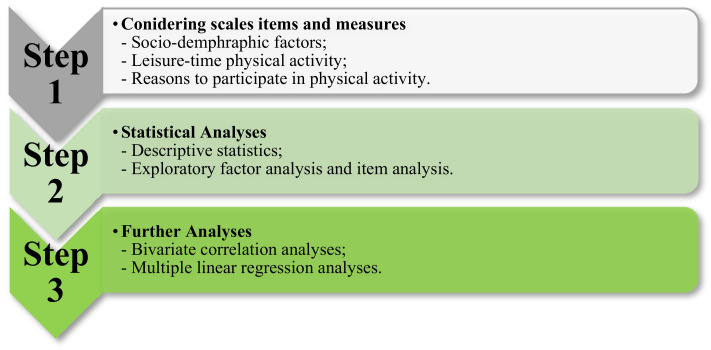
Scale development procedures.

Scales, namely, the Reasons to Participate in Physical Activity Scale (RPPAS), utilized to gauge factors that promote women’s physical activity were developed based on theoretical frameworks from behavioral sciences [28,29,30,31] and existing literature on physical activities [36,37,38,39]. Two experts developed the RPPAS scales (in addition to the author, one professor whose research centers on physical activity and exergaming). Drawing insights from the literature (e.g., [40,41]), we adopted the term “traditional exercise” to refer to an activity requiring physical effort, carried out to sustain or improve health and fitness with little to no dependence on technology, whereas exergaming is defined as video games that require substantial bodily movement to play and function. Participants were recruited to evaluate the preliminary scales. Discrepancies were resolved by rounds of group discussions that involve both the experts and participants until a consensus was reached. The final scale includes 34 factors that shape people’s physical activity. Prior to distribution, the survey was first piloted in a group of individuals who were not familiar with the research to further examine its validity. The RPPAS scale items received positive reviews and were subsequently adopted in the online survey. Detailed information on scale development could be found in Figure 1.

### 2.3. Measures

#### 2.3.1. Sociodemographic Factors

Age and body mass index (BMI) were measured as continuous variables. For the study, race was coded as 1 for Hispanic, 2 for White, 3 for African American, 4 for Asian, and 5 for Others.

#### 2.3.2. Leisure-Time Physical Activity

Participants were asked how often they engaged in vigorous (e.g., running, jogging, hockey, football, soccer, squash, basketball, vigorous swimming, vigorous long-distance bicycling), moderate (e.g., fast walking, baseball, tennis, easy bicycling, volleyball, badminton, easy swimming), and mild exercise (i.e., yoga, archery, fishing from river band, bowling, horseshoes, golf) per week during their leisure time. Leisure-time physical activity was measured by calculating as follows: (Times of vigorous physical activity × 9) + (Times of moderate physical activity × 5) + (Times of mild physical activity × 3) (MET) [42].

#### 2.3.3. Reasons to Participate in Physical Activity

Participants’ motivation for engaging in physical activity was measured by the “Reasons to Participate in Physical Activity” questionnaire developed in the current study. Respondents were asked to rate items such as: “I exercise because it’s a great way to pass time,” “I exercise because it’s an entertaining experience,” and “I exercise because I think it helps me lose weight” on a 7-point scale, from 1 (strongly disagree) to 7 (strongly agree) (Cronbach’s alpha = 0.96).

### 2.4. Statistical Analyses

The analyses for this study were conducted using SPSS 24.0 (IBM SPSS Statistics for Mac, Version 24.0, IBM, Armonk, NY, USA). First, descriptive statistics were conducted and analyzed. Second, exploratory factor analysis and item analysis were performed to examine the preliminary construct validity and internal consistency reliability of the 34 items. Bartlett’s sphericity test and Kaiser’s Measure of Sampling Adequacy were computed to determine the appropriateness of conducting principal components analysis for this data set. Exploratory factor analyses were calculated using principal components analysis with oblique rotation to identify the factor structure. Factor loadings of 0.30 or higher were viewed as acceptable target factor loadings [43]. Finally, bivariate correlation analyses and multiple linear regression analyses were conducted to examine the association of different factors of reasons to participate in physical activity to leisure-time physical activity levels among female college students.

## 3. Results

A total of 108 college female students (M_age_ = 20.34, *SD* = 2.42) were recruited from a large southwestern university. The sample comprised 64 (58.2%) White, 20 (18.2) Hispanic, 16 (14.5%) Asian, 7 (6.4%) African American women, and 3 (2.7%) Others. There were 28 (25.5%) sophomores, 28 (25.5%), juniors (*n* = 63, 21.4%), 28 (25.5%) seniors, 21 (19.1%) freshmen, and 5 (4.5%) postgraduates. The participants’ average of both BMI and leisure-time physical activity were 22.10 ± 2.83 (kg/m^2^) and 52.34 ± 29.84 (MET), respectively.

### 3.1. Exploratory Factor Analysis

The initial exploratory factor analysis was conducted based on a sample of 108 female college students. Several well-recognized criteria for the factor analysis were used. First, it was observed that all 34 items were significantly correlated, suggesting reasonable associations for factor analysis. Second, the Kaiser–Meyer–Olkin measure of sampling adequacy was 0.89, above the commonly recommended value of 0.60 [44], and Bartlett’s test of sphericity was significant (χ2 (561) = 3762.61, *p* < 0.001; Bartlett [45]). The diagonals of the anti-image correlation matrix were also all over 0.80. Finally, the commonalities were all above 0.30 (Table 1), further confirming that each item shared some common variance with other items. Given these overall indicators, it was sufficient to conduct factor analysis with all 34 items.

Principal component analysis was used because the primary purpose was to identify the factors underlying the reason to participate in physical activity. Initial eigenvalues indicated that the first five factors explained 47%, 14%, 7%, 5%, and 4% of the variance, respectively. The solution for the five factors was examined using oblimin rotations of the factor loading matrix. The five-factor solution, which explained 77% of the variance, was preferred based on the scree plot, eigenvalues, and qualitative interpretation. All items in this analysis had primary loading of at least 0.30. The factor loading matrix for this solution is presented in Table 2.

### 3.2. Item Analysis

Internal consistency for each of the items was examined using Cronbach’s alpha. Cronbach’s alpha ≥ 0.70 was considered adequate internal consistency [46]. Factor 1, labeled *enjoyment and gratification*, included 12 items with Cronbach’s alpha = 0.96. Factor 2 was labeled as *Consideration for other activities (i.e., exergaming)* and included five items with Cronbach’s alpha = 0.96. Factor 3, *health benefits*, consisted of five items with Cronbach’s alpha = 0.90. Factor 4, *networking opportunities* included seven items with Cronbach’s alpha = 0.92. Finally, Factor 5, *appearance and performance*, consisted of five items with Cronbach’s alpha = 0.91. The skewness and kurtosis were well within a tolerable range for assuming a normal distribution (Table 3). Overall, these analyses indicated that underlying college female students’ responses were organized into five distinct factors on the reasons to participate in physical activity items and that these factors were strongly internally consistent.

### 3.3. Bivariate Correlation and Multiple Linear Regression

Overall, enjoyment and gratification (*r* = 0.54, *p* < 0.001), consideration for other activities (i.e., exergaming) (*r* = 0.21, *p* < 0.05), health benefits (*r* = 0.26, *p* < 0.01), networking opportunities (*r* = 0.44, *p* < 0.001), and appearance and performance (*r* = 0.49, *p* < 0.001) were significantly associated with leisure-time physical activity. Multiple linear regression analyses were used to examine the association of five different factors to leisure-time PA. The results showed that appearance and performance was uniquely associated with leisure-time PA (*β* = 0.37, *p* = 0.012), after controlling for age, race/ethnicity, and BMI. However, enjoyment and gratification, consideration for other activities (i.e., exergaming), health benefits, and networking opportunities were not associated with leisure-time physical activity.

## 4. Discussion

This paper set out to identify factors that motivate women’s participation in physical activity, insights that could help government and health officials to develop tailored interventions to improve women’s physical activity and health outcomes. This is the first study that examined motivating factors of physical activity that are unique to the female population. Considering the dearth of health measures that are tailored to women’s characteristics and interests [47,48], it is our hope that the current study could inspire more research endeavors that aim to address the concerns and challenges women face in the context of physical activity and beyond. Based on research findings, a scale (i.e., RPPAS) that reflects the unique preferences of women in physical activity was developed. In addition to its women-centeredness, what is also unique about the RPPAS measurement centers on its inclusion of the potential impacts of digital health (i.e., exergaming) on women’s physical activity participation. These research contributions are particularly important amid COVID-19, a critical and high-stake period of time [49] when conditions such as obesity—a prevalent health challenge women face—have further increased women’s susceptibility to COVID-19 infections and deaths [5,6,7].

The main research objective was to investigate factors that motivate women’s participation in physical activity. Drawing insights from the literature [28,29,30,31,36,37,38,39], we developed and validated a 34-item RPPAS scale to examine women’s physical activity behavior in light of digital health considerations such as exergaming. The results from exploratory factor analysis revealed that enjoyment and gratification, consideration for other activities (i.e., exergaming), health benefits, networking opportunities, as well as appearance and performance considerations are considerations that have the potential to promote women’s participation in physical activity. By adding novel insights to the literature, the findings of our study further extend the current understanding of what factors have the potential to promote physical activities in women. 

Interestingly, while all five of these factors are motivations for women’s physical activity, results from multiple linear regression analyses indicate that only consideration for appearance and performance was significantly associated with participants’ physical activity levels after controlling for compounding factors (i.e., age, race/ethnicity, and BMI). Contrary to previous research, which indicates that appearance-related factors negatively impact women’s exercise behaviors [36,37], the current study’s findings underscore the critical role of appearance and performance-based considerations in shaping women’s physical activity levels. Overall, this finding is in line with real-world phenomena seen amid the pandemic. As COVID-19 continues to evolve, emerging evidence shows that, due to factors such as social pressure about physical appearances, women were more likely to experience Zoom fatigue [50]. Ironically, the perceived social pressure, such as the need to wear makeup, above and beyond gender-neutral expectations such as professional attire, has also prompted women, especially women of color, to be more reluctant to return to the office compared to their male counterparts [51,52].

**Figure 2:**
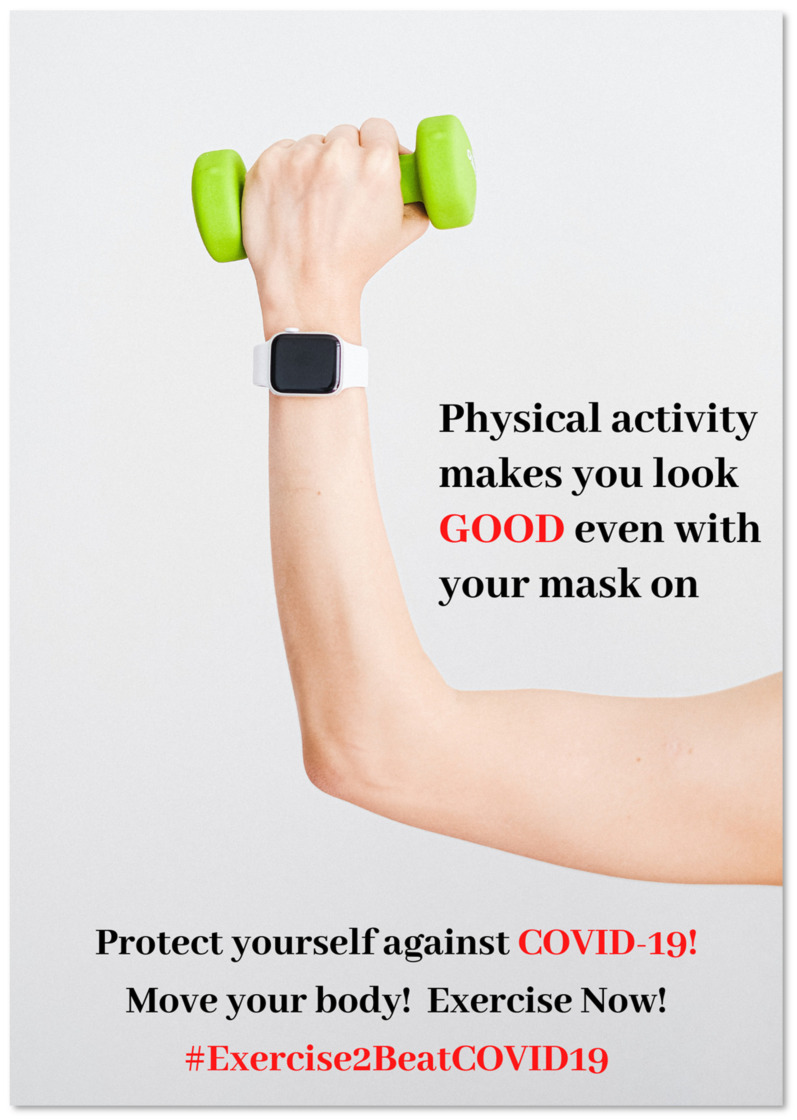
An example physical activity intervention.

**Figure 3:**
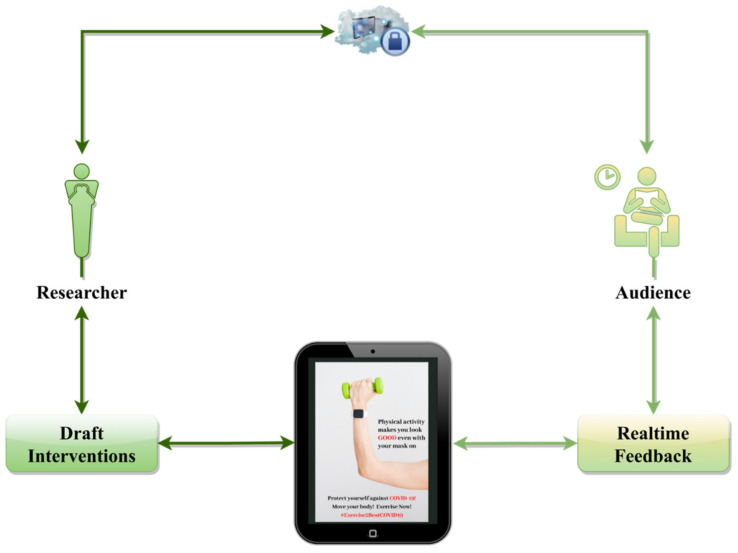
A schematic representation of the proposed co-design process.

In light of these insights, one way to capitalize on the research findings without causing unintended consequences in women is via respectively integrating appearance and performance appeals in physical activity interventions for women, using the co-design method [53]. In other words, researchers should invite women in their design and development of the interventions that are targeted to this population. In light of the constraints posed by the pandemic, such as limitations on in-person meetings [54], another way to adopt the co-design method in developing women-specific physical activity interventions is via developing a theory-guided and evidence-based campaign and then gaining the key target audience’s insights [55], such as the example campaign the authors developed in the current study (see Figure 2). A schematic representation of the proposed co-design model could be found in Figure 3. Overall, this iterative design method could include all stakeholders in deciding what the “final” representation of the intervention should be (e.g., Does the intervention representative of all body sizes and skin colors?), in light of its effectiveness and appropriateness.

People’s physical activity has been substantially disrupted by COVID-19, particularly due to safety mandates such as lockdowns and social distancing [56,57,58,59]. In a study of 2524 Italian adults, researchers found that both men and women’s physical activity significantly decreased amid the pandemic, the reduction of which is associated with the deterioration of participants’ mental well-being [57]. Research on 2002 adults in the United Kingdom further shows that individuals with higher BMI and lower physical activity are more likely to face mental health issues amid lockdowns [15]. While there are challenges for people to access facilities such as gyms during the pandemic, there are substantial benefits associated with physical activity amid COVID-19 [16,17,18,19].

In a study of 2850 Spanish adults, for instance, findings show that amid shelter-at-home mandates, participants who have greater physical activity experience lower levels of anxiety and mood swings [18]. Overall, these insights combined underscore the important implications of this study in informing physical activity intervention design and development for young women amid COVID-19. Future studies could explore ways to further leverage the importance of appearance and performance considerations for young women in physical activity interventions amid the pandemic, with the aim to alleviate potential physical and mental health challenges young women face during COVID-19.

Another reason why participatory design methods such as the co-design approach proposed in this study are important centers on the interplay between mental health and physical activity, particularly among women [60]. Existing evidence suggests that women often experience a greater scale and scope of mental health challenges, which could have an adverse impact on their participation in physical activity as well as overall health [61,62]. Mental health issues may also have a negative impact on women’s interpretation of self-image, along with considerations for appearance and performance (e.g., weight stigma), which may further compound women’s physical activity participation [63,64,65,66]. The compounding impact of the pandemic should not be overlooked [67]. A study on mental health issues caused by COVID-19 further indicated that the pandemic has resulted in an additional 53.2 million cases of major depression and 76.2 million cases of anxiety disorders globally, both of which are more prevalent in women than men [68]. These insights, combined, paired with the potential added impacts of the Omicron variants [69], underscore the need to ethically and morally leverage the findings of the study—striving for optimal intervention outcomes without causing unintended consequences in women, as it is fundamental that interventions that aim to help do not incur harm.

### Limitations

While this research fills important gaps in the literature, it is not without limitations. First, the survey developed in this study is self-administered by the participants, which suggests that results are subject to social desirability and recall biases. Second, this present study is cross-sectional in nature, indicating that findings are limited in their causal implications. Third, the sample size of the study is small, which could further impact the rigor of the research findings. Future research could address these limitations by inviting large and diverse female populations to participate in the study, preferably utilizing the longitudinal design, to further enrich the literature. Another limitation of our study is that we have yet to conduct a study to gauge the construct validity of the developed scale. We plan to address this limitation in our follow-up investigations.

## 5. Conclusions

A world without healthy women is a world without a promising future. Obesity and lack of physical activity are compromising women’s abilities to care for themselves and their loved ones, as well as their capabilities to contribute to society. This study set out to identify factors that motivate women to participate in physical activity. Our findings underscored the importance of factors such as consideration for appearance and performance in shaping women’s physical activity. Furthermore, the study results also emphasized the need for a nuanced understanding of influences that shape women’s physical activity levels, ranging from concerns for enjoyment and gratification, consideration for other activities (i.e., exergaming), and health benefits to networking opportunities. Future research could investigate how to leverage these motivators in tailored health interventions that aim to improve women’s physical activity.

## Figures and Tables

**Table 1:** Descriptive statistics of all female and male participants.

Variable	Total (%)
**N**	403 (100.0)
**Age** (years, Mean ± SD)	20.2 (±2.3)
**Gender**	
Male	108 (26.8)
Female	295 (73.2)
**Race**	
White	235 (58.3)
Non-White	168 (41.7)
**Income**	
~USD 19,999	83 (21.0)
USD 20,000~USD 74,999	103 (26.0)
USD 75,000~	210 (53.0)
**BMI** (kg/m^2^, Mean ± SD)	22.3 (±3.1)
**Leisure-time physical activity** (MET; Mean ± SD)	57.5 (±56.1)

**Table 2:** Factor loadings and communalities for 34 items from RPPAS.

Items	Factor 1	Factor 2	Factor 3	Factor 4	Factor 5	Communalities
4. Doing exercise gives me pleasure.	0.92					0.84
6. I consider myself as an avid exerciser.	0.87					0.80
2. I exercise to unwind.	0.85					0.81
3. I exercise because it’s an entertaining experience.	0.84					0.77
7. Being good at doing exercise has become an identity to me.	0.81					0.77
1. I exercise because it’s a great way to pass time.	0.79					0.62
9. I exercise because it’s addictive.	0.76					0.69
5. I like to escape in my exercise.	0.75					0.77
8. I feel related to other people when I exercise.	0.58					0.63
10. I feel I’m in control when doing exercise.	0.49					0.70
20. I take pride in my exercise skills.	0.43					0.76
17. Doing exercise gives me a sense of accomplishment.	0.30					0.64
32. I exercise and play active games at the same time because I want to improve my exercise performance in active gaming.		0.96				0.92
33. I exercise to learn more about active gaming skills and methods.		0.93				0.93
31. I exercise because I like the active gaming and want to play more of it.		0.92				0.87
34. I exercise to work on my form for active gaming techniques.		0.91				0.87
30. I exercise to improve my sports skills in active gaming.		0.85				0.78
27. For me, traditional exercise costs less, compared to other forms of exercise (e.g., active gaming), which may require certain devices or gadgets.			0.94			0.84
28. Doing traditional exercise is a quicker form of exercise than other exercise methods (e.g., active gaming).			0.91			0.82
29. I exercise because they fit better into my schedule over other exercise methods (e.g., active gaming).			0.76			0.66
26. Traditional exercise offers more varieties of easily accessible exercise choices than other forms of exercise (e.g., active gaming).			0.61			0.77
25. I exercise because it’s more convenient, compared to other exercise methods (e.g., active gaming).			0.54			0.77
14. I think competing in various exercise methods with my friends/family strengthens our relationships.				0.88		0.81
18. I like to compete against my friends/family while doing exercise.				0.86		0.83
13. I always have a quality time with my friends/family when we were doing exercise.				0.82		0.66
19. I like to dominate other players when doing exercise.				0.81		0.75
12. I exercise because it’s a great way to stay close to my friends/family.				0.75		0.62
16.Competing/winning while doing exercise gives me great self-satisfaction.				0.74		0.63
15. I exercise because I want to excel at certain exercise practice.				0.45		0.72
23. I exercise because I think it helps me lose weight.					0.97	0.83
22. I exercise because I think it helps me stay fit.					0.87	0.89
24. Doing exercise improves my appearance.					0.87	0.85
11. Doing exercise makes me feel I’m constantly making progress.					0.47	0.77
21. I constantly try to reach new goals when doing exercise.					0.47	0.71

*Note.* Factor loadings < 0.3 are suppressed. Factor 1 = Enjoyment and gratification; Factor 2 = Consideration for other activities (i.e., exergaming); Factor 3 = Health benefits; Factor 4 = Networking opportunities; Factor 5 = Appearance and performance. PA = physical activity.

**Table 3:** Descriptive statistics from exploratory factor analysis of the five factors (*N* = 108).

Factor	No. of Items	*M* (*SD*)	Skewness	Kurtosis	Cronbach’s α
Factor 1	12	4.33 (1.56)	−0.52	−0.64	0.96
Factor 2	5	2.10 (1.59)	1.55	1.51	0.96
Factor 3	5	4.91 (1.51)	−0.78	0.32	0.90
Factor 4	7	3.66 (1.58)	−0.001	−0.87	0.92
Factor 5	5	5.48 (1.42)	−1.51	2.24	0.91

*Note.* Factor 1 = Enjoyment and gratification; Factor 2 = Consideration for other activities (i.e., exergaming); Factor 3 = Health benefits; Factor 4 = Networking opportunities; Factor 5 = Appearance and performance. PA = physical activity. *M* = mean; *SD* = standard deviation.

## Data Availability

Data are available upon request.

## Abbreviations

BMI	body mass index
IRB	Institutional Review Board
RPPAS	Reasons to Participate in Physical Activity Scale
